# Antiretroviral Therapy as a Factor Protective against Anal Dysplasia in HIV-Infected Males Who Have Sex with Males

**DOI:** 10.1371/journal.pone.0092376

**Published:** 2014-03-27

**Authors:** Carmen Hidalgo-Tenorio, Mar Rivero-Rodriguez, Concepción Gil-Anguita, Mercedes Lopez De Hierro, Pablo Palma, Jessica Ramírez-Taboada, Javier Esquivias, Miguel Angel López-Ruz, Rosario Javier-Martínez, Juan Pasquau-Liaño

**Affiliations:** 1 Infectious Disease Unit, University Hospital Virgen de las Nieves, Granada, Spain; 2 Gastroenterology Service, University Hospital Virgen de las Nieves, Granada, Spain; 3 General Surgery Service, University Hospital Virgen de las Nieves, Granada, Spain; 4 Pathology Service, University Hospital Virgen de las Nieves, Granada, Spain; The Chinese University of Hong Kong, Hong Kong

## Abstract

**Objectives:**

Chronic infection with oncogenic HPV genotype is associated with the development of anal dysplasia. Antiretroviral therapy (ART) has been shown to decrease the incidence of cervical carcinoma in women with HIV. We sought to: 1) describe the prevalence and grade of anal dysplasia and HPV infection in our study subjects; 2) analyze the grade of correlation between anal cytology, PCR of high-risk HPV, and histology; 3) identify the factors associated with the appearance of ≥AIN2 lesions.

**Design:**

Cross-sectional, prospective study.

**Methods:**

A cohort of HIV-positive males (n = 140, mean age  = 37 years) who have sex with males (MSM) had epidemiological, clinical and analytical data collected. Anal mucosa samples were taken for cytology, HPV PCR genotyping, and anoscopy for histological analysis.

**Results:**

Within the cohort, 77.1% were being treated with ART, 8.5% anoscopy findings were AIN2, and 11.4% carcinoma *in situ*; 74.2% had high-risk (HR), 59.7% low-risk (LR) HPV genotypes and 46.8% had both. The combination of cytology with PCR identifying HR-HPV better predicts the histology findings than either of these factors alone. Logistic regression highlighted ART as a protective factor against ≥AIN2 lesions (OR: 0.214; 95%CI: 0.054–0.84). Anal/genital condylomas (OR: 4.26; 95%CI: 1.27–14.3), and HPV68 genotype (OR: 10.6; 95%CI: 1.23–91.47) were identified as risk factors.

**Conclusions:**

In our cohort, ART has a protective effect against dysplastic anal lesions. Anal/genital warts and HPV68 genotype are predictors of ≥AIN2 lesions. Introducing PCR HPV genotype evaluation improves screening success over that of cytology alone.

## Introduction

Over the last few decades there has been an increase in the incidence of anal cancer due, in large part, to the increase in risk groups such as males who have sex with males (MSM), the immunocompromised and, especially, patients with HIV infection [1].

The incidence of anal cancer in HIV-positive patients varies between 40 and 137 per 100,000 person/years, which is much higher than in the general seronegative population, with a predominance of males with AIDS staging [2], [3]. One of the principal risk factors associated with the appearance of this neoplasm is the chronic infection with high-risk genotypes of the human papilloma virus (HR-HPV), also termed oncogenic genotypes [4]. This infection in HIV patients is favored by specific factors such as low levels of CD4 [5], previous chlamydia infection, smoking habit [6] or being MSM [1]. Anal cancer has a considerable similarity with cancer of the cervix in terms of the different stages and natural history of the disease, such as the initial process of acquiring the HPV infection, followed by the persistence of the virus in the mucosa that favors the progression to high-grade anal intraepithelial neoplasia (HGAIN) and, subsequently, to invasive cancer [7]. Studies conducted in HIV-positive patients show a progression in the premalignant lesions of anal carcinoma from AIN1 to AIN2-3 in 12.8 cases/1000 patient-months [8]. In seronegative patients, the progression from AIN1 to AIN 2-3 is estimated at 62% within approximately 24 months, and that the progression from AIN3 to invasive carcinoma is around 9-13% within 5 years [9].

Anal cancer in HIV patients is currently considered one of the most-frequent non-AIDS-defining malignancies [2]. Several studies confirm its greater prevalence compared to the seronegative population and the appearance, according to the majority of authors, has not diminished despite anti-retroviral therapy (ART) [10], [11], [12], [13]; except in the case of the Swiss cohort study in which a reduction of this neoplasia was detected around the beginning of the late period of ART between the years 2002–2006 [14]. Along the same lines, there have been 2 studies on the benefits of ART in HIV-MSM patients not only in the progression of the pre-malignant anal lesions (derived from a Canadian cohort), but also in the prevalence of these lesions (derived from a Dutch transversal study) [15]. Finally, of note is that ART in HIV-positive women has demonstrated reduction in the incidence of cancer of the cervix [16], regression of dysplastic lesions, and elimination of the HPV genotypes [17].

Hence, we proposed as principal objective: 1) to describe the prevalence and grade of anal dysplasia, and of the infection by HPV, in our cohort of HIV-positive MSM patients; and as secondary objectives: 2) to analyze the degree of correlation between anal cytology and PCR of HR-HPV with the histology findings from biopsy and 3) to study the factors associated with the appearance of ≥AIN2 lesions in our cohort. The factors analyzed included that of ART.

## Patients and Methods

### Design

Cross-sectional study conducted between May 2010 and Sept. 2013 in a cohort of 140 patients with HIV-MSM recruited consecutively into a program of screening, diagnosis, treatment and follow-up of dysplastic lesions of the anal mucosa. The HIV-positive patients were from among those receiving attention in the Infectious Disease Unit of the *Hospital Universitario Virgen de las Nieves* (Granada, Spain) the ethics committee of which approved the study.

The inclusion criteria were: adult (≥18 years of age) MSM infected with HIV. The exclusion criteria were: females, heterosexual HIV-positive males, and history of anal canal neoplasia at the time of recruitment into the study.

The objectives and conditions of the study were explained to the patients at the first clinical visit, and written informed consent was obtained (as outlined in PLoS consent form). Clinical, epidemiological and analytical data were obtained and codified for anonymity according to the laws on protection of personal information currently in existence in Spain.

The variables collected were: age, history of perianal or genital condylomatosis, number of different sexual partners in the previous 12 months, condom use in sexual intercourse, tobacco use, alcohol use (standard units of consumption; SUC), intravenous drug abuse (IDA), HIV acquisition route, months since HIV diagnosis, HIV stage according to the CDC Atlanta; months on ART, virologic treatment failure (when the viral mRNA was >50 copies/mL in at least 2 measurements within the previous 6 months), use of concomitant treatments, other infections including chronic hepatitis B and C (HBV and HCV, respectively), luetic positive serum (syphilis), other sexually transmitted diseases (STD), latent tuberculosis or tuberculosis under treatment or active infection.

The laboratory analytes measured included: lymphocyte number, CD4 nadir, CD4, CD8, and viral load at the time of HIV diagnosis together with CD4, CD8 and viral load at the time of inclusion into the study.

At the clinical visit, 2 mucosa samples were taken from the anal canal with cotton swabs soaked in physiologic saline, and stored in liquid medium (thin layer liquid) for the detection and genotyping of the HPV using the polymerase chain reaction (PCR) technique (GeneAmp PCR System 9700, Applied Biosystems, Roche), and for cytology using the ThinPrep Pap Test (Thin Prep Processor 2000, Hologic Corp). Both samples were sent to the anatamo-pathology laboratory where the same senior pathologist of the research team (JE) carried out the cytology evaluation, validation of the PCR, and histology analyses. The genotypes 16, 18, 26, 31, 33, 35, 39, 45, 51–53, 56, 58, 59, 66, 68, 73 and 82 were considered high risk (HR-HPV). Genotypes 6, 11, 34, 40, 42–44, 54, 55, 57, 61, 70–72, 81, 83, 84 and 89 were considered low risk (LR-HPV) [18].

Subsequently, anoscopy by the digestive tract specialist of the research team (MLdeH) was performed within an interval of between 4 and 12 weeks from the cytology assessment. A standard endoscope of 9 mm with working channel of 2.8 mm was used, without any image enhancement. A short exploration of 15–20 cm was performed with retrovision maneuver to better visualize the pectinea line. Samples were taken for histology examination using an endoscopic retrograde cholangio-pancreatography (ERCP) catheter not only from the lesions (color change with irrigation with 5% acetic acid), but also from other parts of the quadrant with ostensibly-normal mucosa.

The cytology classification was that of Bethesda [19] which classifies the lesions into 3 types: atypical squamous cells (ASC), low-grade squamous intraepithelial lesions (LSIL) and high-grade squamous intraepithelial lesions (HSIL).

The histology classification employed divides the lesions into LSIL (AIN1/condyloma), HSIL (AIN2, AIN3/Carcinoma *in situ*), and invasive carcinoma [20]. We considered lesions ≥AIN1 those that spanned AIN1 to carcinoma *in situ*. We considered lesions ≥AIN2 those that proceeded beyond AIN2 to carcinoma *in situ*.

### Statistical analyses

#### Sample size

To achieve a precision of 8% in the calculation of a rate using Normal asymptotic bilateral confidence interval of 95% assuming that the prevalence of infection and of any grade of anal dysplasia is 70%, it would be necessary to include 126 subjects in the study. With an expected loss of 10%, it would be necessary to recruit 140 patients.

#### Descriptive analyses

The general description of the principal variables included central tendency and dispersion (mean, standard deviation, median, percentiles) for the quantitative variables, and the absolute frequencies with 95% confidence intervals (95%CI) for the qualitative variables. The prevalence and 95%CI were calculated for HPV, dysplasias obtained from cytology, and dysplasias obtained from the histology evaluation. Diagnostic success, not only from cytology but also from PCR of HR-HPV of dysplasias that included lesions ≥AIN1 and ≥AIN2, was defined by the receiver operating characteristics (ROC) curves. The results were considered poor: 0.5 to 0.6, acceptable: 0.6 to 0.75, good: 0.75 to 0.9, very good: 0.9 to 0.97, and excellent 0.97 to 1. The degree of concordance between cytology, PCR of HR-HPV, and biopsy results was analyzed using the Kappa index. The results of the test were evaluated using the classification of Landis and Koch in which a value of k<0.20 would be considered poor; 0.21 to 0.40 weak; 0.41 to 0.60 moderate; 0.61 to 0.80 good; and 0.81 to 1.00 very good [21].

#### Bivariate analyses

We used bivariate analyses to assess the relationship between the possible risk factors and the presence of dysplastic lesions ≥AIN2. The Student *t*-test for independent samples was applied for quantitative variables that followed a normal distribution, while the Mann-Whitney test was employed for those variables that did not follow normal distributions. The Kolmogorov-Smirnov test was used to assess whether the different variables fulfilled the criteria of normal distribution. Comparison of differences between variables was with the Pearson χ^2^ test, or the Fisher exact test if the application criteria were not fulfilled.

#### Multivariate analyses

Logistic multivariate regression was applied based on the classic formula of Freeman [n = 10*(k+1)], [22]. Included in the model were the results that were statistically significant in the bivariate analyses, as well as those considered clinically relevant. Variables were introduced into the analyses manually one by one while leaving out those variables that did not modify the model so as, finally, to construct a model with those variables that did have a modifying effect (ART, current perianal condylomas, clinical history of syphilis, HPV 68, AIDS stage, duration of HIV, duration of ART, number of HR-HPV genotypes in anal mucosa, CD4 nadir, CD4 nadir <200 cells/uL). A method of selection using successive steps was employed considering, in each step, a probability of entry of 0.05 and that of 0.10 for exit. The Hosmer-Lemeshow test was employed to assess the goodness of adjustment for the logistic regression model.

A value of p<0.05 was considered statistically significant in all the analyses. The statistical software used was the SPSS (version 15.0).

## Results

Outcomes data and details of methods are available from the corresponding author, on request.

### 1. Characteristics of the cohort of HIV-MSM patients

Included were 140 patients, mean age 37 years, CD4 nadir 356 cells/μL, median time of clinical evolution of the HIV infection around 33 months (IQR: 11–84), 78% on treatment with combination anti-retroviral therapy (ARTc) over a median period of 23.5 months at which time there were 652 cells/μL CD4, and only 6% in virologic treatment failure The characteristics are summarized in Table 1.

**Table 1:** General description of the patient cohort, and results of the PCR of HPV, cytology and anoscopy.

Characteristics	Patients MSM-HIV; n = 140
Mean age; years (± SD)	37.27 (±8.9)
Nationality: Spanish; n (%)	133 (95)
Retired; n (%)	8 (5.8)
Education level; n (%)	
University	73 (52.2)
Secondary school	47 (33.6)
Primary school	19 (13.6)
None	1 (0.7)
Median number of partners over previous 12 months (IQR)	1 (1–6.75)
Habitually using condoms; n (%), (95%CI)	109 (77.9), (71–81)
Perianal/genital condylomatosis; n (%), (95%CI)	42 (30), (20–36)
History of condylomas; n(%), (95%CI)	50 (35.7), (28–44)
Median duration of HIV; months, (IQR)	33 (11–84)
Mean VL of HIV (log), (±SD)	3.83 (±4.33)
CD4 (cells/μL), (±SD)	652.87 (±261.71)
CD8 (cells/μL), (±SD)	1431.07 (±4366.35)
CD4 nadir (cells/μL), (±SD)	356.29 (±246.92)
AIDS stage (A3, B3, C); n (%), (95%CI)	46 (32.9), (27–43)
Receiving ART; n (%), (95%CI)	108 (77), (70–84)
Number of months of ART; mean (IQR)	23.5 (8–80.3)
Virological treatment failure; n (%), (95%CI)	7 (6), (2–10)
History of syphilis treated; n (%), (95%CI)	26 (18.6), (12–26)
Other STD; n (%), (95%CI)	56 (40), (32–50)
Latent tuberculosis treated; n (%)	17 (12.5)
Chronic HCV infection; n (%)	6 (4.3)
Chronic HBV infection; n (%)	3 (2.1)
Smoking habit; n (%), (95%CI)	67 (47.9), (41–58)
Ex-IVDA, n (%)	2 (1.5)
Median daily alcohol consumption; (SAU), (IQR)	0 (0–1)

MSM: males who have sex with males; VL: viral load; HCV: hepatitis C virus infection; HBV: hepatitis B virus infection; SAU: standard alcohol units; ex-IVDA: ex-intravenous drug abuser; SD: standard deviation; STD: sexually transmitted diseases; IQR (inter-quartile range).

### 2. Results of the cytology, PCR of the HPV and anal histology

We found that more than half the patients had dysplasia, 49.2% had LSIL, 2.5% and HSIL and 2.5% had ASC. Of the cohort, 88.6% were HPV-positive according to PCR, 74.2% (95%CI: 69–85) of whom had high-risk genotype, 59.7% (95%CI: 51–70) low-risk genotype and 46.8% (95%CI: 39–58) both genotypes. The HPV genotypes isolated most frequently in anal mucosa were: 16 (30.6%), 6 (16.1%), 51 (15.3%), 84 (13.7%), 11 (12.9%), 18 (12.1%), and 61 (12.1%) (Table 2). Of the 140 anoscopies performed, only 32.8% had normal histology, the rest having some grade of dysplasia, of which 20% had lesions ≥AIN2, and 11.4% (95%CI: 4–19) with carcinoma *in situ* (Table 2).

**Table 2.** Results of the PCR of HPV, cytology and anoscopy.

Outcomes		MSM-HIV patients; n = 140
PCR of HPV-positive, n (%), 95%CI		124 (88.6)
	High-risk HPV	74 (59.7), (51–70)
	Low-risk HPV	92 (74.2), (69–85)
	Low and High-risk HPV	58 (46.8), (39–58)
Genotypes, n (%)	HPV16	38 (30.6)
	HPV6	20 (16.1)
	HPV51	19 (15.3)
	HPV 84	17 (13.7)
	HPV11	16 (12.9)
	HPV18	15 (12.1)
	HPV 61	16 (12.1)
Anal cytology; n (%), 95%CI		120 (85.7)
	Normal	55 (45.8), (39–57)
	LSIL	59 (49.2), (38–57)
	ASC	3 (2.5), (0–6)
	HSIL	3 (2.5), (0–6)
Anoscopy; Histology, n (%), 95%CI	Normal	46 (32.8), (28–44)
	AIN1	66 (4.1), (38–56)
	AIN2	12 (8.5), (4–13)
	AIN3/Carcinoma *in situ*	16 (11.4), (4–19)

HPV: human papilloma virus; HR-HPV: high-risk human papilloma virus, LR-HPV: low-risk human papilloma virus; LSIL: low-grade squamous intraepithelial lesion; HSIL: high-grade squamous intraepithelial lesion; ASC: atypical squamous cells; AIN: anal intraepithelial neoplasia. IQR (inter-quartile range).

**Figure 1:**
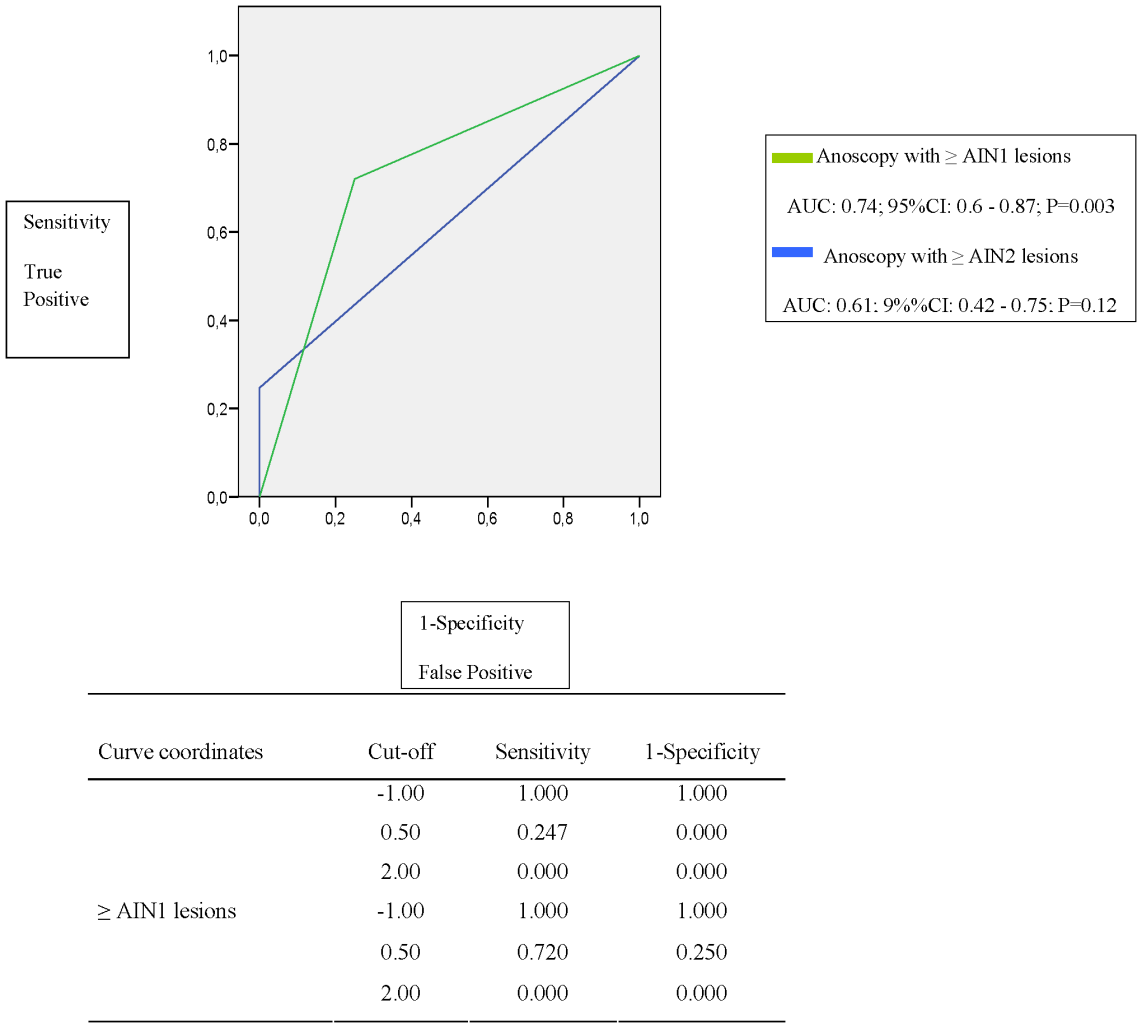
ROC curve of HR-HPV-negative and normal cytology *vs.* ≥AIN 1 and ≥AIN 2 lesions.

**Figure 2:**
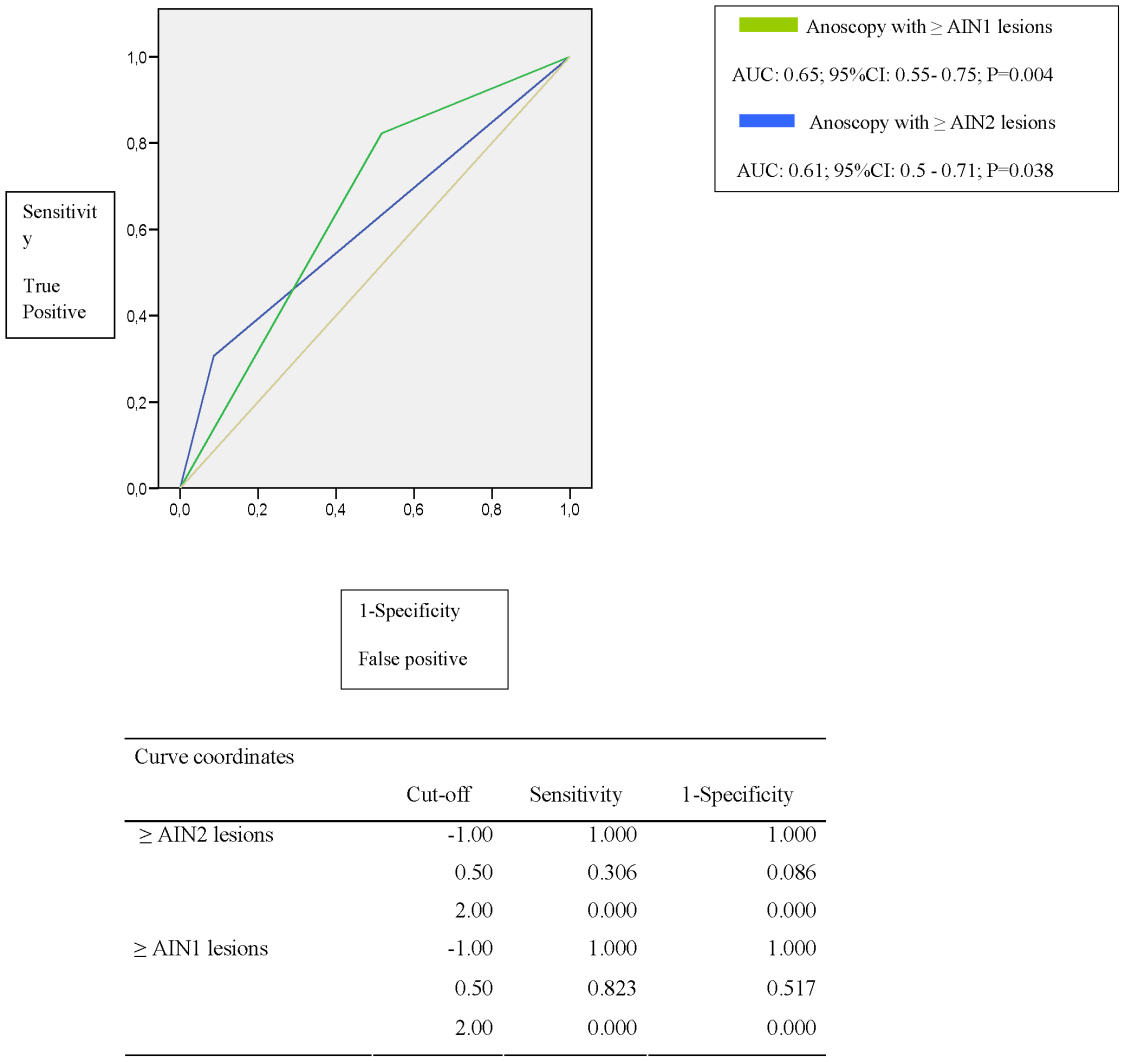
ROC curve of normal cytology *vs*. ≥AIN 1 and ≥ AIN 2 lesions.

The diagnostic performance curves of the cytology, and those from PCR of HR-HPV genotypes used to predict the presence of dysplasia in the histology, revealed acceptable results. The area under the curve (AUC) values of the receiver operating characteristics (ROC) were not clearly discriminatory; the majority of values being around 6. The combination of HR-HPV PCR with cytology better predicts the grade of dysplasia than the cytology alone i.e. in those with normal cytology and HPV-negative PCR, the AUC of the ROC was 0.74 (p = 0.003) while, in the case of normal cytology alone, it was 0.65 (p = 0.04) (Figures 1 and 2).

Correlations between the cytology results and PCR of HR-HPV were poor when each was considered independently, in relation to the histology of the anal mucosa. Of note is that only 48.3% of the patients with normal cytology had a normal biopsy; the rest of the patients presented anal dysplasia such as carcinoma *in situ* (3.4%) and AIN2 (5.2%). However, when we analyzed cytology and PCR of the HR-HPV in combination, the diagnostic efficiency was considerably improved. In case of normal cytology with PCR of HR-HPV-positive, 24.4% presented carcinoma *in situ*/AIN3 and 6.8% AIN2 i.e. 31.1% had lesions ≥AIN2. Of further note is that in case of normal cytology with PCR of HR-HPV-negative, 75% of the patients had normal histology, and none had lesions ≥AIN2 (Table 3)

**Table 3:** Degree of correlation between anal cytology and HR-HPV PCR, with histology

Variable	Normal histology	AIN1	AIN2	AIN3/Carcinoma *in situ*	≥AIN2 lesions
	n (%); Kappa; p*	n (%); Kappa; p*	n (%); Kappa; p*	n (%); Kappa; p*	n (%) ; Kappa; p*
Normal cytology (n = 58)	28 (48.3)	25 (43.1)	3(5.2)	2 (3.4)	5 (8.6)
	0.31; 0.0001	-0.15; 0.1	-0.46; 0.35	-0.11;0.04	-0.22; 0.003
ASC (n = 3)	0 (0)	3 (100)	0 (0)	0 (0)	0 (0)
	0.49; 0.22	0.048; 0.08	0.04; 0.6	-0.41;0.57	0.47;0.38
LSIL (n = 59)	11 (18.6)	34 (57,6)	5 (8.5)	13 (2.03.3)	18 (30.1)
	0.27; 0.001	013; 0.14	0.02; 0.6	0.19;0.02	0.21; 0.005
HSIL (n = 3)	0 (0)	2 (66.7)	1 (33.3)	0 (0)	1 (33.3)
	0.02; 0.22	0.02; 0.5	0.12; 0.08	-0.41;0.57	0.3; 0.56
HR-HPV positive (n = 92)	29 (31.5)	45 (48.9)	7 (7.6)	14(15.29)	21 (22.8)
	-0.17; 0.01	0.13; 0.83	-0.03; 0.4	0.07;0.07	0.04; 0.39
Normal cytology	9 (20)	25 (55.5)	3 (6.8)	11 (24.4)	14 (31.1)
+ HR-HPV positive (n = 45)	-0.27; 0.005	0.14; 0.15	-0.03; 0.6	0.22; 0.002	0.19; 0.029
Normal cytology	12 (75)	4(25)	0	0	0
+ HR-HPV-negative (n = 16)	0.3; 0.0001	-0.14; 05	0.12; 0.19	-0.12;017	-0.21;0.03

Kappa: Kappa index; p* significance.

### 3. Risk factors associated with the appearance of ≥AIN2 lesions

In the bivariate analysis of the different factors associated with the appearance of lesions ≥AIN2, we observed, as a risk variable, the number of HR-HPV genotypes in anal mucosa (p = 0.02) especially genotype 68 (p = 0.01). Longer-term ART was a protective factor (p = 0.02), as was a longer time since HIV diagnosis (p = 0.007) (Table 4).

**Table 4:** Risk factors associated with the appearance of lesions ≥AIN2. Univariate analysis.

	HIV-MSM with ≥AIN2 lesions; n = 28	HIV-MSM without ≥AIN2 lesions; n = 112	P*
Age; mean years (±SD)	35.1(±8.2)	37.8(±8.9)	0.149
Nationality: Spanish, n (%)	26 (92.9)	107 (95.5)	0.63
Retired; n (%)	1 (3.6)	7 (6.3)	1
University education; n (%)	17 (60.7)	56 (50)	0.31
Partners previous year; median (IQR)	1 (1–9.5)	1 (1–6)	0.4
Condom use, n (%)	21 (75)	88 (78.6)	0.7
Perianal/genital condylomatosis, n (%)	12 (42.9)	30 (26.7)	0.09
History of condylomas, n (%)	9 (32.1)	41 (36.7)	0.7
Duration of HIV; mean months (IQR)	18.5 (3–35.3)	36 (14.25–84)	0.007
VL of HIV log10 (copies/mL) (± SD)	4.03(±4.52)	4.23(±3.2)	0.3
CD4 mean (cell/uL), (± SD)	627.4(±289.4)	659.8(±254.9)	0.57
CD8 mean (cell/uL), (± SD)	967.8(±403.8)	1558.7(±4924.6)	0.54
CD4 mean nadir (cell/uL), (± SD)	354.5(±232)	356.8(±251.9)	0.96
AIDS stage (A3, B3, C), n (%)	6 (21.4)	40 (34.5)	0.15
ART, n (%)	17 (69.7)	89 (79.5)	0.02
Median duration of ART; months (IQR)	14.5 (0.25–27)	28.5 (10–87.5)	0.008
Virological treatment failure, n (%)	1 (4.5)	6 (6.4)	1
Syphilis treated, n (%)	4 (14.3)	22 (19.6)	0.5
Latent tuberculosis treated, n (%)	4 (14.3)	13 (11.6)	0.7
HCV, n (%)	2 (7.14)	4 (3.6)	0.5
HBV, n (%)	1 (3.6)	2 (1.8)	0.34
Smoking habit, n (%)	14 (50)	53 (47.3)	0.8
PCR of HPV			
LR-HPV, n (%)	18 (69.2)	56 (57.1)	0.3
HR-HPV, n (%)	21 (80.8)	71 (72.4)	0.4
HR+LR HPV, n (%)	14 (53.8)	44 (44.9)	0.4
Number of HR-HPV (IQR)	2 (1–3.25)	1 (0–2)	0.2
Number of LR-HPV (IQR)	1 (0–2)	1 (0–2)	0.8
Genotypes, n (%)			
HPV6	7 (26.7)	13 (13.2)	0.1
HPV11	6 (23.1)	10 (10.2)	0.1
HPV16	11 (42.3)	27 (27.5)	0.2
HPV18	6 (23.1)	9 (9.2)	0.08
HPV51	6 (23.1)	13 (12.2)	0.2
HPV 61	6 (23.1)	10 (10.2)	0.1
HPV 68	5 (19.2)	3 (13.2)	0.01
HPV 84	4 (4.1)	13 (13.2)	0.7

MSM-HIV-positive: males who have sex with males HIV-positive, VL: viral load; HCV: hepatitis C virus; HBV: hepatitis B virus; HPV: human papilloma virus; VL: HIV viral load; HR-HPV: high risk human papilloma virus; LR-HPV: low risk human papilloma virus; IQR: inter-quartile range.

Finally, in the logistic regression analysis assessing appearance of lesions ≥AIN2, we identified the risk factors as: the presence of perianal condylomas during the study (OR: 4.26; 95%CI: 1.27–14.3) and the HPV genotype 68 (OR: 10.6; 95%CI: 1.23–91.47) whose prevalence was around 6.5%. The factors protective against the appearance of these lesions were identified as: receiving ART (OR: 0.21; 95%CI: 0.054–0.84), and having had a previous diagnosis of syphilis (OR: 0.078; 95%CI: 0.008–0.72).

## Discussion

Our cohort of HIV-positive MSM patients had a high prevalence not only of dysplastic lesions (50% LSIL in cytology and 20% biopsy lesion ≥AIN2) but also infection by high-risk HPV genotypes of around 74%. The results were very similar to those published by other authors who had observed the percentage of anal dysplasia with low grade cytology (LSIL) at around 40%, 19% ≥AIN2 lesions and the most-frequently identified HPV16 genotype [23].

The prevalence of anal carcinoma *in situ*/AIN3 in our group of patients, was 11.4% (95%CI: 4–19) which translates into 1/9 patients having anal cancer *in situ*. AIN2 was 8.5%, i.e. 1/5 of patients included had pre-malignant lesions (HSIL). These findings highlight the need to implement routine consultations in HIV clinics which, currently in Spain, are not performed systematically for screening, diagnosis, treatment and follow-up of dysplastic lesions of the anal mucosa. Anal cancer is one of the most frequent non-AIDS defining malignancies in HIV, and has become so probably because of the increase in survival of HIV patients [24]. Nevertheless, there has not been any consensus nor homogeneity of recommendations by the different scientific societies regarding the screening or treatment of anal dysplasia in HIV-positive patients [25], [26], [27].

The HPV genotype most frequently identified in the anal mucosa of our patient cohort was the oncogenic 16, present in about 30.6% of the patients included in this study. It is one of the most frequent, high-risk genotypes isolated in anal mucosa of HIV-positive MSM patients [28], as well as in HIV-negative males [29], HIV-positive women [30] and heterosexual HIV-positive men [31].

The correlation between the results of the cytology and histology that we observed are slightly more encouraging; only 48.3% of the patients with normal cytology had a normal biopsy (Kappa index: 0.31; p = 0.0001). Of the rest, 43.1% were AIN1, 5.2% AIN2 and 3.4% carcinoma *in situ*. Conversely, when the patient with normal cytology had high-risk HPV in anal mucosa, only 20% had a normal histology. Of the rest, 55.5% had AIN1, 6.8% AIN2 and 24.4% AIN3/carcinoma *in situ*. Of further note is that in patients with normal cytology with PCR of high-risk HPV-negative, 75% had normal histology, and none had ≥AIN2 lesions. Our results have some important consequences for recommendations for screening of premalignant lesions and carcinoma of the anal canal in HIV patients. To date, the different AIDS scientific societies recommend only anal cytology [25], [26], [27]. This would fail to diagnose, in our cohort for example, 1/3 patients with ≥AIN2 lesions. Hence, apart from the cytology in screening for dysplastic lesions of anal mucosa, we recommend the performance of HPV PCR. In case of normal cytology and HR-HPV-negative, anoscopy may be avoided and a new check-up scheduled for the next year, and to include cytology and PCR. In case the cytology was dysplastic or the HR-HPV PCR was positive, anoscopy would be performed. Also, the current recommendations of annual cytology assessments alone to discard diagnosis of AIN1 lesions would, as well, not be appropriate. The few studies that have addressed this issue in follow-up have detected progression to premalignant lesions in 62% within approximately 24 months, and progression from AIN3 to carcinoma of between 9% and 13% in 5 years [9].

We observed the presence of anal condylomatosis as well as HPV68 genotype as being risk factors associated with the appearance of ≥AIN2 lesions. Genital-anal condylomatosis is due to the infection by the HPV genotypes and, similar to those that present in the anal canal mucosa, are associated with the appearance of dysplasia not only in women [32] but also in men [33]. Hence, it can be considered a factor predictive of lesions ≥AIN2. As such, it would seem highly necessary to recommend, in HIV-positive MSM patients with genital and or perianal warts, that they are screened for anal dysplasia. Despite that genotype HPV16 was the most-frequently isolated in our cohort, it is the high-risk HPV68 that is associated with the appearance of ≥AIN2 lesions. This genotype has oncogenic capacity belonging to the C group of the phylogenetic tree of the papilloma virus, similar to genotypes 18, 39, 45 and 59 [34]. These genotypes, as opposed to the HPV16 in patients with similar immunological background, could more successfully avoid immune vigilance and give rise to dysplastic lesions in the anal mucosa [23].

We observed that receiving ART and previous diagnosis of luetic infection were factors predisposing against the appearance of ≥AIN2 lesions. With respect to ART, we support the findings of the Swiss study [14] and the probable underlying mechanism could be similar to that occurring in the cervix i.e. modulation of the oncogenic effect of the HR-HPV by maintaining suppression of the HIV replication. This reduction in HIV viral load in anal mucosa would reduce HPV replication and, subsequently, favor the clearance of the HPV. Also, our results support the findings of Pokomandy et al who observed, in a prospective cohort study of 3 years duration with 247 HIV-positive MSM. The patients received ART for >4 years and obtained a benefit against the appearance of AIN2-3 lesions [18] while, conversely, the low levels of CD4 as well as the presence of HPV genotypes 16/18 were the principal factors associated with progression. Finally, the data from a cross-sectional study in Holland composed of 250 HIV-positive MSM patients showed a lower prevalence of AIN2/3 lesions and HPV infection in those receiving ART compared to those who were not. As such there was a higher prevalence of these lesions in those with HPV infection; essentially genotypes 6 and 16 [15].

That previous infection with *Treponema pallidum* can be a protective factor against the appearance of ≥AIN2 lesions, could be interpreted as syphilis being a surrogate marker for early diagnosis of HIV and, thus, of the infection by HPV which is encountered in an earlier phase in which the appearance of the dysplastic lesions have not, as yet, occurred.

Finally, a weak point of our study needs to be addressed. The study was cross-sectional and, as such, no causal relationships between the measured variables could be demonstrated. The strength of the study is the consistency of the data. All the anoscopies, biopsy and cytology of anal mucosa, as well as the PCR of HPV, microbiology laboratory assessments, and clinical interviews were each performed by the relevant specialist, and this minimizes inter-observer bias.

In conclusion, in our cohort of HIV-positive MSM patients, 3/4 were infected with HPV genotypes of high-risk; 1/5 had high-grade lesions. There was a low correlation between the cytology findings and the anal histology. Adding PCR of the HPV genotypes to the screening for ≥AIN2 lesions would increase the diagnostic efficiency above that of cytology alone. Antiretroviral treatment could exercise a protective role against the presence of lesions ≥AIN2. The presence of genital or perianal warts and HPV68 genotype in anal mucosa are 2 factors predictive of ≥AIN2 lesions. These findings warrant further investigation.
